# Assessment of the Effect of Soil Sample Preparation, Water Content and Excitation Time on Proximal X-ray Fluorescence Sensing

**DOI:** 10.3390/s22124572

**Published:** 2022-06-17

**Authors:** Shuo Li, Jiali Shen, Thomas F. A. Bishop, Raphael A. Viscarra Rossel

**Affiliations:** 1 Key Laboratory for Geographical Process Analysis & Simulation of Hubei Province, Central China Normal University, Wuhan 430079, China; shenjienie@mails.ccnu.edu.cn; 2 School of Life & Environmental Sciences, Sydney Institute of Agriculture, The University of Sydney, 1 Central Avenue, Australia Technology Park, Eveleigh, NSW 2015, Australia; thomas.bishop@sydney.edu.au; 3 Soil & Landscape Science, School of Molecular & Life Sciences, Faculty of Science & Engineering, Curtin University, GPO Box U1987, Perth, WA 6845, Australia; r.viscarra-rossel@curtin.edu.au

**Keywords:** XRF spectroscopy, soil elemental composition, fine-earth fraction, soil moisture effect, scan time, proximal sensing

## Abstract

X-ray fluorescence (XRF) spectroscopy offers a fast and efficient method for analysing soil elemental composition, both in the laboratory and the field. However, the technique is sensitive to spectral interference as well as physical and chemical matrix effects, which can reduce the precision of the measurements. We systematically assessed the XRF technique under different sample preparations, water contents, and excitation times. Four different soil samples were used as blocks in a three-way factorial experiment, with three sample preparations (natural aggregates, ground to ≤2 mm and ≤1 mm), three gravimetric water contents (air-dry, 10% and 20%), and three excitation times (15, 30 and 60 s). The XRF spectra were recorded and gave 540 spectra in all. Elemental peaks for Si, K, Ca, Ti, Fe and Cu were identified for analysis. We used analysis of variance (anova) with post hoc tests to identify significant differences between our factors and used the intensity and area of the elemental peaks as the response. Our results indicate that all of these factors significantly affect the XRF spectrum, but longer excitation times appear to be more defined. In most cases, no significant difference was found between air-dry and 10% water content. Moisture has no apparent effect on coarse samples unless ground to 1 mm. We suggested that the XRF measurements that take 60 s from dry samples or only slightly moist ones might be an optimum option under field conditions.

## 1. Introduction

As a laboratory-based analytical method, X-ray fluorescence (XRF) has become increasingly popular and been adopted by environmental consultancies, research institutions, and governmental agencies due to the need for environmental monitoring to address current concerns over food, water and energy security, land degradation and climate change [1]. The XRF technique relies on the fluorescence at specific energies of excited atoms when irradiated with X-rays. Detection of the specific fluorescent photons enables the qualitative and quantitative analysis of the elements in a sample.

Given the technological advances in recent years, portable XRF devices have developed and improved dramatically with several significant advantages, including being practical, rapid, non-destructive, cost-efficient, and allowing for the identification of many elements simultaneously [2,3,4]. In the laboratory, the use of XRF spectroscopy has traditionally focused on either the elemental composition of soils or heavy-metal contamination, and can do so accurately [5,6,7,8]. Though plenty of studies in the literature on XRF have been successful in using the approach on field-condition samples [9,10,11,12,13,14,15,16], many studies have been carried out under laboratory conditions to obtain better analytical performance via the preparation of homogeneous samples (dried, crushed, ground and sieved). The XRF spectral data obtained under field conditions can significantly differ from laboratory conditions. There are three main interfering factors that might simultaneously influence XRF spectra’s performance and thereby decrease the estimation accuracy of the elemental concentrations detected by XRF spectroscopy; they are surface roughness of soil specimens, soil water content and excitation time.

Packed samples or compacted powders are used as specimens in the laboratory. To reduce sample-to-sample particle size variability, dried soil samples are usually ground to pass through a 2 mm sieve, which would parallel common soil preparation for particle size analysis as well as many other chemical extractions and micro-plate assays [17]. For instance, 0.5 mm [18], 0.15 mm [6] and 200 μm particle size [5] were also used by other studies. As outlined in Markowicz [19], soil samples with a large number of fine particles tend to generate higher concentrations of the analyte when compared to samples containing larger particles despite equal concentrations of the analyte of interest.

The water content in soil causes fluorescence attenuation, leading to a lower precision, poor detection limit and lower accuracy. When the water content is between 5% and 20%, the overall error may be minimal [20]. Drying of the sample is needed because data can be unreliable when the soil moisture content is above 20% [10,21]. In contrast, Zhang et al. [16] recommended that samples containing <30% water content do not need to be corrected for moisture.

**Figure 1:**
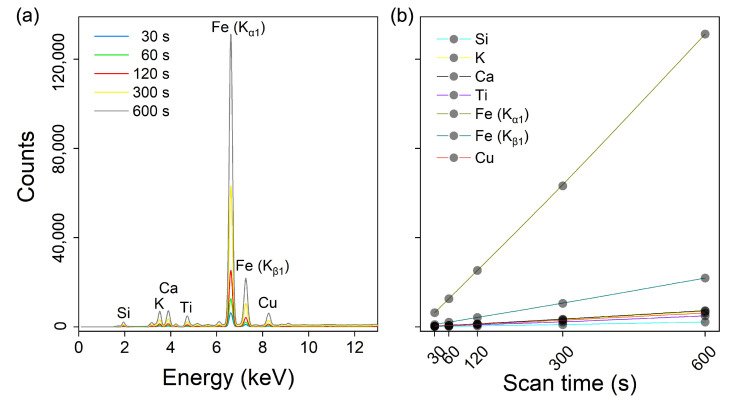
Representative XRF soil spectrum annotated with some dominant peaks and their corresponding energies (**a**) and the changes in the character peaks’ intensity under the excitation times changes (**b**).

Of these three factors, excitation time is the only one that can be manipulated during scanning. A longer time will give a ‘smoother’ appearance to the XRF spectrum, which typically improves the detection limit and the measurement precision by reducing relative uncertainties. The XRF intensity of the peak increases accordingly with the count time (see Figure 1). Shorter excitation times (e.g., 10–30 s) are widely used by XRF [5], though longer (≥2 min) ones have also been adopted [18,22]. As summarised by Tighe et al. [23], longer times will reduce detection limits only up to a point before the signal-to-noise ratio becomes sub-optimal.

Despite extensive literature directed toward the prediction of soil properties using XRF spectral data, we still are unclear of the interaction effects of different sample preparations, water contents and excitation times on the soil XRF spectra. The answer to this question is essential for an investigator to use XRF technique for cost-effective [24] and reliable [25] soil analysis. We therefore aim to investigate and discuss these individuals and their interactions. The ambition is not to propose a way to overcome such effects on XRF analysis of soils, but to help XRF users.

## 2. Materials and Methods

### 2.1. Factorial Design

**Figure 2:**
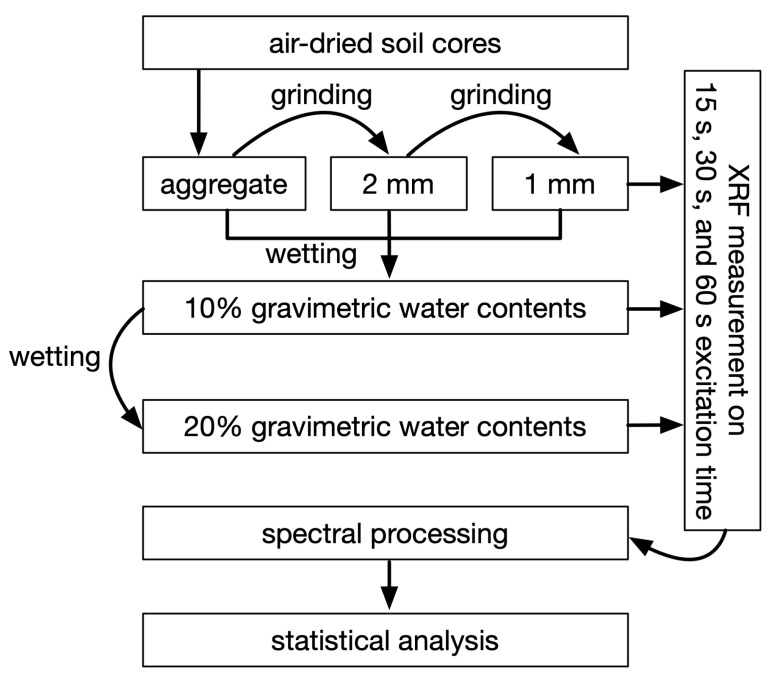
Flowchart of the methodology.

We implemented a three-way factorial design with blocking. The treatment factors were three sample preparations (natural aggregates and ground to ≤2 mm and ≤1 mm); three gravimetric water contents (air-dry, 10% and 20% on a weight basis); and three excitation times (15, 30 and 60 s). Sixty seconds was chosen as the upper limit as this is the maximum amount of time we want to spend on one sample or at a single location for future work in the field. Figure 2 shows the flowchart that describes the steps in data preparation, collection, and evaluation.

### 2.2. Physicochemical Analysis and Sample Preparations

We randomly selected four samples from four intact soil cores which had previously undergone laboratory analyses (see Table 1). The soil cores were sampled with a hydraulic soil corer to a depth of 1 m into 50 mm diameter PVC liners. A small section of each core was selected based on clay content to provide four soils with a wide range of clay contents. After oven-drying at 40 °C for 48 h, a number of the dried natural soil aggregates were set aside for further analysis. The remains were ground, sieved to ≤2 mm, and subset into two parts––one for further analysis, the other were fine-ground to pass a 1 mm sieve. Thus, we had three levels of sample preparations: aggregate, ≤2 mm and ≤1 mm. Five sub-samples were collected and homogenized by hand mixing for each ground sample.

### 2.3. Water Measurements

Soil water content was measured gravimetrically after the XRF spectra of air-dried samples were measured. Samples were weighed to calculate 10% gravimetric water content. Each sample was wet to the determined level before being scanned again. This rewetting process was then repeated but for 20% gravimetric water content. Thus, three levels of gravimetric water content were obtained: air-dry, 10% and 20%.

### 2.4. Spectroscopic Measurements

We scanned all samples using a portable XRF analyzer system (Amptek, Bedford, MA, USA). The system consists of an X-ray tube system and a spectrometer. The Mini-X X-ray tube contains a grounded anode with variable current and voltage controlled via USB as an excitation source. It features a 50 kV/80 μA power supply, a silver transmission target, and a beryllium end window. The spectrometer consists of a high-resolution Si-PIN photodiode mounted on a thermoelectric cooler, and the input field-effect transistor (FET) coupled to a customs charge sensitive preamplifier. The thermoelectric cooler reduces the electronic noise in the detector and preamplifier. We implemented routine calibration on the spectrometer before measurement. The XRF spectra were produced from 1024 energy channels (keV) in this study.

**Figure 3:**
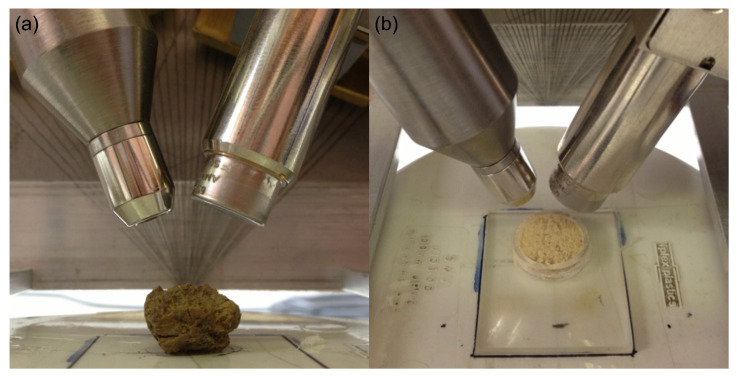
The portable XRF analyzer and spectral measurement for (**a**) natural soil aggregate and (**b**) 1 mm ground soil sample.

Air-dry aggregate samples were placed underneath the focal point of the spectrometer for spectral measurement (Figure 3a). Ground soil samples were placed in a 10 mm diameter by 3 mm-thick plastic sample holder without compression and levelled by a gentle tap (Figure 3b). To reduce noise and to enhance the signals, five scans were averaged into one spectrum for each sample to be used as a replicate in the anova.

### 2.5. Spectral Processing and Analysis

After their scans, spectra were smoothed with a local regression smoothing function in the software R [26] using the *pavo* package [27]. A span of 0.01 was chosen as it removed spectral noise while preserving the original spectral shape. Several clear visible peaks could be found from each spectrum. A total of six (Si, K, Ca, Ti, Fe(K${}_{\alpha }$) and Cu) characteristic elemental peaks were identified for investigating the response of the XRF spectrum. Their full width at half height (FWHH) and the maximum height (H${}_{\max}$) were obtained from the spectrum. Their peak areas were calculated by multiplying H${}_{\max}$ by FWHH.

An analysis of variance (anova) and post hoc tests were applied to identify the significant differences in mean peak intensity and area across all three factors: sample preparation, water content and excitation time. The peak intensity and area data were log-transformed before analysis to meet the assumption of normality. A significance level of *p* < 0.05 according to unprotected Fisher’s Least Significant Difference (LSD) test was considered to be statistically significant. We used GenStat [28] for data analysis.

## 3. Results and Discussion

### 3.1. Description of Spectral Peaks Intensity and Area

A summary statistic of six elemental peaks’ intensity and area is displayed in Table 2. Due to the higher fluorescence energy (see Figure 1), Fe(K${}_{\alpha }$) at 6.4 keV had the highest peak intensity, area and greatest range of values (from 27 to 407). Conversely, Ca at 3.7 keV had the lowest peak intensity, area and the smallest range of values (from 1 to 4), owing to intense self-absorption.

### 3.2. Performance of Excitation Time on XRF Spectra

**Figure 4:**
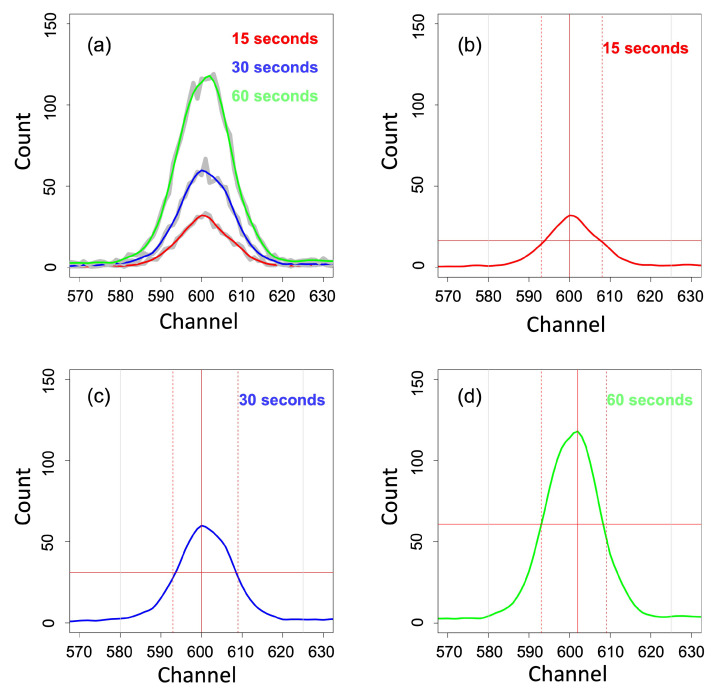
Processed XRF spectra and peak data extraction. (**a**) XRF spectra of Fe(K${}_{\alpha }$${}_{1}$) peak at 15, 30 and 60 s excitation times (grey lines) and smoothed spectra (coloured lines). (**b**–**d**) Plot from ‘peakshape’ function for Fe(K${}_{\alpha }$${}_{1}$) peak at 15, 30 and 60 s excitation times, respectively. The vertical continuous red line indicates the peak location, the horizontal continuous red line indicates the half-maximum count (or intensity), and the distance between the dashed lines is the full width at half height (FWHH).

Scanned under excitation times of 15, 30, and 60 s, the spectra of all soils were similar, with some characteristic peaks apparent. Taking Fe(K${}_{\alpha }$) for example, Figure 4a shows the result of the application of smoothing spectra with various excitation times. Both raw spectra (grey line) and processed spectra (coloured line) are similar but distinct. We can see that the noise, especially at the apex of the peak, has been removed. A visual representation of this process is displayed for the Fe(K${}_{\alpha }$) peak shown in Figure 4d for 15, 30 and 60 s excitation times, respectively. In general, and as expected, the data derived from increased excitation times led to a more pronounced peak and increase in area.

### 3.3. Performance of Sample Preparation and Water Content

**Figure 5:**
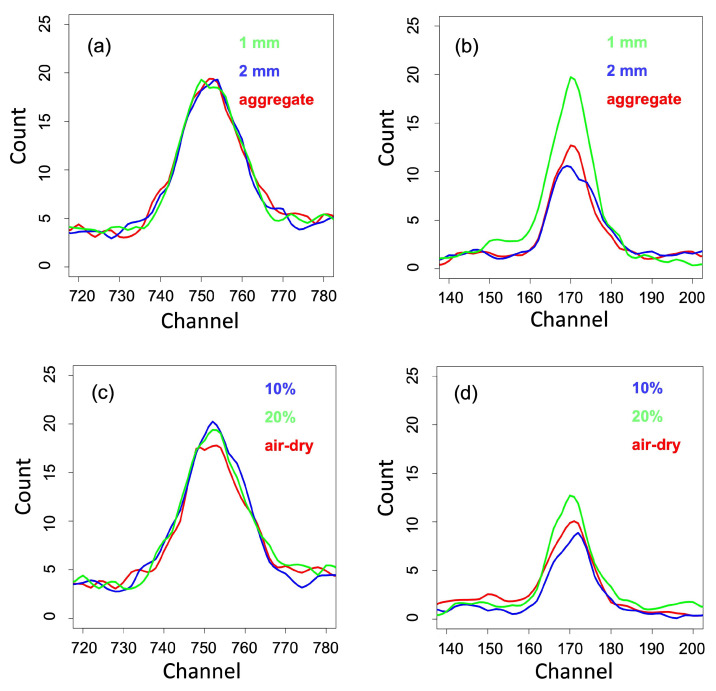
Difference in XRF spectral response of soil as a result of sample preparations and water content treatments for Cu (**a**,**c**), and Si (**b**,**d**). (**a**,**b**) are showing the effect of sample preparations while (**c**,**d**) are showing the effect of water content.

Except for Cu, there was no apparent difference among different particle sizes (see Figure 5a). Its air-dry spectra have the lowest value at the apex of the peak, and both spectra on 10% and 20% water contents have no clear difference (Figure 5c). In most cases, however, the ≤1 mm particle size and the 20% water content resulted in the highest intensity at the apex of peaks (e.g., Si shown in Figure 5b,d).

### 3.4. Analysis of Variance

Table 3 shows the result of the effects of ‘water content’, ‘sample preparation’, and ‘excitation time’, as well as all two- and three-way interactions on both peak intensity and peak area of all six elements. In the case of the three-way interaction effect, all of the *F* values were small, and the *p* value was greater than 0.05 (last row of Table 3). Therefore, we can examine the two-way interaction effects.

The results of two-way anova interactions (middle part in Table 3) showed that there were no significant interactive effects between sample preparation or water content with the excitation time (P × T or W × T) for these six elements. We only found significant interactive effects between the sample preparation and water content (P × W) on the peak intensity of Si, K, Ca, and Fe, but no significance in terms of peak area, except for Fe. Without understanding both the physical mechanism of the XRF matrix effect and the nature of the soil heterogeneity effect, such interaction behaviours of different elements can be challenging to interpret.

In the case of the main effects, which were not involved in a significant two-way interaction, all had a significant effect except for on Cu, for which there was no significant difference for sample preparations in intensity and peak area, and for moisture content, there was no difference in intensity. For ease of interpretation, the main effects not involved in a significant interaction are italicised.

Tjallingii et al. [29] found that heavier elements (such as K, Ca, Ti and Fe) are relatively unaffected by soil moisture, but those lighter elements (e.g., Si) are more sensitive to moisture changes, which is not consistent in our findings. There is no consensus about which elements are more influenced by soil moisture and how much water can influence the XRF analysis. Santana et al. [30] found that elements with the highest atomic number (such as Cu) were clearly impacted by water content, but Ti and Fe were not. In some Brazilian soil samples, Si was much more influenced by moisture than Ti and Fe [31].

### 3.5. Post Hoc Tests

Table 4 presents the significant two-way interactions, where the LSD (Least Significant Difference) results are shown by the superscripted letters. Fine grinding to 1 mm gives the most considerable value of intensity and area.

Except for Si and Fe, in terms of 10% and 20% water content, respectively, sample preparation had no significant effect unless when fine grinding to 1 mm, which might be because it gives the biggest value of intensity and area. Therefore, moisture has no effect in most cases unless we grind to 1 mm.

On the other hand, for aggregate soil or soil samples ground to ≤2 mm, water content had no significant effect unless when wetter (20%), excluding Si. However, such insignificance on 20% water content disappeared at a finer particle size (1 mm). Hence, sample preparation has no effect in most cases unless using a wetter sample e.g., 20% water content.

The summarised results of the LSD tests on the main effects are displayed in Table 5. We used ‘–’ to represent those involved in a significant two-way interaction.

As the water content increased from air-dry to 10% and to 20%, values did not show a consistent liner relationship in either the peak intensity or area or both for the five elements (see Table 5). This is because the water in soils absorbs the characteristic X-rays of the analytes and produces stronger scattering of the primary radiation, which leads to a reduction in the characteristic X-ray intensities and an increase in the scattered X-ray intensities [32].

As water has a significant effect on Cu in peak area (see Table 3), there was a significant difference between wet soil and dry soil in terms of peak area. For Si, each water level has a significant difference in terms of the area. However, for K, Ca, and Ti, no significant difference was found between air-dry and 10% water content, but when the soil was wetter, a significant difference was found between 20% with 10% water content or air-dry soil, which was consistent with the findings of the two-way interaction (Table 4). Goff et al. [17] attributed this to fluorescence attenuation.

In general, the water in soil significantly affects the XRF spectrum when the content is higher than 10%. We suggested that soil samples with moisture levels above 10% should be dried prior to analysis with XRF spectra. The methods to correct for moisture influence, e.g., in Refs. [21,31,33], might be considered when working in field conditions.

From aggregate to 2 mm and further to 1 mm (Table 5), overall, values of either the peak intensity or area decreased a little bit then increased and surpassed that of air-dry soil. This was expected due to the relatively smooth surface of the aggregate sample and enrichment of radiation in the smallest particles.

A significant difference was found between ≤1 mm particle size with ≤2 mm and aggregate soil in peak intensity or area, except for Cu. In contrast, there was no significant difference for K, Ca and Cu in peak intensity and area between ≤2 mm particle size and the aggregate soil, while peak intensity and area for Ti were significantly different. Because fine grinding is time-consuming, soil without grinding could be considered in XRF work, with caution for Si and Ti.

All levels of excitation times showed significant differences from each other for all six elements. As expected, a scan length of 60 s produced significantly higher peak intensity and area. As shown in Figure 4, the peaks produced from the longer scan times also appeared to be more defined, with readings becoming more stable with increased scan length.

Silva et al. [34] indicated that excitation times between 30 and 60 s are usually sufficient for screening-level analysis. Although we did not show any quantitative analysis results in this work (e.g., using XRF spectra to estimate Cu concentrations), our anova results demonstrated that the excitation time has a significant effect on the XRF spectrum, and we suggest 60 s as an optimal option for using the portable XRF.

## 4. Conclusions

We found that ‘water content’, ‘sample preparation’, and ‘excitation time’ had various influences on the XRF spectrum. There was no clear tendency or significant difference between light and heavier elements. As might be expected, longer excitation times generally improved detection limits, where a strong linear relationship exists between the intensity of an XRF spectrum and excitation times. Excitation time had the most significant effect on signal intensity, while sample preparation and water had secondary effects. The results of the anova varied for each of the six characteristic elemental peaks that we analysed. Interactive effects on the peak intensities of Si, K, Ca, and Fe were identified only between sample preparation and water content.

In most cases, sample preparation had little effect on the measurements when the sample was wet. Measurements of dry samples or only slightly moist ones that take 60 s might represent an optimum option when measuring with a portable XRF spectrometer under field conditions. More work is needed on wet samples (e.g., water content ≥ 20%), which would complicate the analysis of XRF for proximal soil sensing.

## Figures and Tables

**Table 1:** Summary statistics of the laboratory analysis for soil samples.

Sample	Depth	pH	Clay	Silt	Sand	TOC	EC
	cm		%	%	%	%	μS cm${}^{-1}$
Sample 1	30	7.99	53.26	26.63	19.99	0.39	125.5
Sample 2	5	5.02	19.14	29.13	51.93	1.88	171.3
Sample 3	10	5.06	30.79	37.45	31.84	2.44	273.4
Sample 4	30	5.17	7.49	23.30	69.12	0.22	117.8

TOC = total organic carbon, EC = electrical conductivity.

**Table 2:** Statistical description of the mean elemental peak intensity and area (in keV) for 108 soil samples.

Statistics	Si		K		Ca		Ti		Fe (K${}_{\alpha }$)		Cu	
	int.	Area	int.	Area	int.	Area	int.	Area	int.	Area	int.	Area
Min.	2	26	4	58	1	14	5	64	27	453	4	61
Med.	6	73	12	181	4	70	12	177	114	1841	10	175
Mean	7	97	14	224	4	79	14	208	139	2236	11	199
Max.	25	325	33	493	11	204	32	463	407	6447	23	371
Std dev.	4.8	63.1	7.9	126.6	2.5	46.3	7.6	115.5	89.3	1437	6.0	103.5
Skew.	1.3	1.3	0.7	0.6	0.8	0.6	0.6	0.5	1.0	1.0	0.4	0.4

int. = intensity, Min. = minimum, Med. = median, Max. = maximum, Std dev. = standard deviation, Skew. = skewness.

**Table 3:** Results (*F* values) of two-way and three-way anova on the effects of the water content (W), sample preparation (P) and excitation time (T), and their interactions on the peak intensity and peak area of XRF spectrum.

Source	Si		K		Ca		Ti		Fe		Cu	
	int.	Area	int.	Area	int.	Area	int.	Area	int.	Area	int.	Area
Main effects												
W	*61.83* *	41.53 *	*40.11* *	5.84 *	*12.12* *	7.08 *	33.81 *	24.67 *	*20.26* *	*19.06* *	1.78	9.34 *
P	*226.27* *	171.02 *	*103.60* *	18.75 *	*94.66* *	34.23 *	128.45 *	108.16 *	*35.13* *	*33.82* *	2.93	1.96
T	1099.12 *	1005.33 *	2618.59 *	1420.14 *	1984.64 *	651.21 *	3146.30 *	2755.89 *	1606.19 *	1415.69 *	3144.56 *	1878.04 *
2-way Interactions												
W × T	0.60	0.24	0.13	0.77	2.77	1.84	0.50	0.13	0.07	0.02	2.28	0.43
P × T	0.60	0.96	0.87	0.82	2.48	0.28	1.74	0.81	0.14	0.09	0.64	1.61
P × W	4.49 *	1.74	3.38 *	1.38	7.00 *	1.21	1.89	2.18	5.51 *	5.13 *	0.92	0.85
3-way Interactions												
P × W × T	0.77	0.39	1.09	0.56	0.71	0.63	1.20	0.55	0.06	0.05	0.39	0.26

* = *p* < 0.05; values given in italics means the main effects not involved in a significant interaction.

**Table 4:** The post hoc (LSD) pairwise comparisons of sample preparation and excitation time.

Level		Si	K	Ca	Fe	
**P**	**W**	**int.**	**int.**	**int.**	**int.**	**Area**
aggregate	air-dry	1.638 ${}^{c}$	2.347 ${}^{a}$	1.138 ${}^{a}$	4.64 ${}^{a}$	7.406 ${}^{a}$
2 mm		1.665 ${}^{c}$	2.326 ${}^{a}$	1.153 ${}^{a}$	4.584 ${}^{a}$	7.341 ${}^{a}$
1 mm		2.159 ${}^{f}$	2.601 ${}^{c}$	1.438 ${}^{c}$	4.852 ${}^{b}$	7.629 ${}^{b}$
aggregate	10%	1.504 ${}^{b}$	2.339 ${}^{a}$	1.139 ${}^{a}$	4.571 ${}^{a}$	7.358 ${}^{a}$
2 mm		1.379 ${}^{a}$	2.323 ${}^{a}$	1.157 ${}^{a}$	4.611 ${}^{a}$	7.414 ${}^{a}$
1 mm		2.053 ^e^	2.607 ${}^{c}$	1.499 ${}^{c}$	4.832 ${}^{b}$	7.625 ${}^{b}$
aggregate	20%	1.861 ${}^{d}$	2.528 ${}^{b}$	1.314 ${}^{b}$	4.81 ${}^{b}$	7.578 ${}^{b}$
2 mm		1.774 ${}^{d}$	2.508 ${}^{b}$	1.291 ${}^{b}$	4.787 ${}^{b}$	7.585 ${}^{b}$
1 mm		2.221 ${}^{f}$	2.664 ${}^{c}$	1.425 ${}^{c}$	4.844 ${}^{b}$	7.636 ${}^{b}$
aggregate	air-dry	1.638 ${}^{c}$	2.347 ${}^{a}$	1.138 ${}^{a}$	4.64 ${}^{a}$	7.406 ${}^{a}$
	10%	1.504 ${}^{b}$	2.339 ${}^{a}$	1.139 ${}^{a}$	4.571 ${}^{a}$	7.358 ${}^{a}$
	20%	1.861 ${}^{d}$	2.528 ${}^{b}$	1.314 ${}^{b}$	4.81 ${}^{b}$	7.578 ${}^{b}$
2 mm	air-dry	1.665 ${}^{c}$	2.326 ${}^{a}$	1.153 ${}^{a}$	4.584 ${}^{a}$	7.341 ${}^{a}$
	10%	1.379 ${}^{a}$	2.323 ${}^{a}$	1.157 ${}^{a}$	4.611 ${}^{a}$	7.414 ${}^{a}$
	20%	1.774 ${}^{d}$	2.508 ${}^{b}$	1.291 ${}^{b}$	4.787 ${}^{b}$	7.585 ${}^{b}$
1 mm	air-dry	2.159 ${}^{f}$	2.601 ${}^{c}$	1.438 ${}^{c}$	4.852 ${}^{b}$	7.629 ${}^{b}$
	10%	2.053 ^e^	2.607 ${}^{c}$	1.499 ${}^{c}$	4.832 ${}^{b}$	7.625 ${}^{b}$
	20%	2.221 ${}^{f}$	2.664 ${}^{c}$	1.425 ${}^{c}$	4.844 ${}^{b}$	7.636 ${}^{b}$

LSD = Least Significant Difference; a–f different letters between rows indicate a significant difference (*p* < 0.05).

**Table 5:** Results of post hoc tests used to establish whether main effects of sample preparations, water content and excitation times affected XRF spectral response.

Factors	Level	Si		K		Ca		Ti		Fe		Cu	
		int.	Area	int.	Area	int.	Area	int.	Area	int.	Area	int.	Area
Water content	air-dry	–	4.421 ${}^{b}$	–	5.205 ${}^{a}$	–	4.145 ${}^{a}$	2.431 ${}^{a}$	5.144 ${}^{a}$	–	–	2.277 ${}^{a}$	5.093 ${}^{a}$
	10%	–	4.236 ${}^{a}$	–	5.242 ${}^{a}$	–	4.136 ${}^{a}$	2.427 ${}^{a}$	5.129 ${}^{a}$	–	–	2.296 ${}^{a}$	5.162 ${}^{b}$
	20%	–	4.498 ${}^{c}$	–	5.294 ${}^{b}$	–	4.27 ${}^{b}$	2.549 ${}^{b}$	5.249 ${}^{b}$	–	–	2.265 ${}^{a}$	5.182 ${}^{b}$
Sample preparation	aggregate	–	4.269 ${}^{b}$	–	5.203 ${}^{a}$	–	4.129 ${}^{a}$	2.419 ${}^{b}$	5.131 ${}^{b}$	–	–	2.275 ${}^{\mathrm{ab}}$	5.156 ${}^{a}$
	2 mm	–	4.189 ${}^{a}$	–	5.199 ${}^{a}$	–	4.053 ${}^{a}$	2.366 ${}^{a}$	5.064 ${}^{a}$	–	–	2.261 ${}^{a}$	5.121 ${}^{a}$
	1 mm	–	4.697 ${}^{c}$	–	5.339 ${}^{b}$	–	4.368 ${}^{b}$	2.622 ${}^{c}$	5.327 ${}^{c}$	–	–	2.302 ${}^{b}$	5.16 ${}^{a}$
Excitation time	15 s	1.172 ${}^{a}$	3.74 ${}^{a}$	1.805 ${}^{a}$	4.557 ${}^{a}$	0.612 ${}^{a}$	3.448 ${}^{a}$	1.794 ${}^{a}$	4.482 ${}^{a}$	4.04 ${}^{a}$	6.829 ${}^{a}$	1.612 ${}^{a}$	4.479 ${}^{a}$
	30 s	1.775 ${}^{b}$	4.351 ${}^{b}$	2.475 ${}^{b}$	5.236 ${}^{b}$	1.279 ${}^{b}$	4.22 ${}^{b}$	2.484 ${}^{b}$	5.183 ${}^{b}$	4.725 ${}^{b}$	7.497 ${}^{b}$	2.266 ${}^{b}$	5.148 ${}^{b}$
	60 s	2.47 ${}^{c}$	5.063 ${}^{c}$	3.135 ${}^{c}$	5.947 ${}^{c}$	1.96 ${}^{c}$	4.883 ${}^{c}$	3.129 ${}^{c}$	5.857 ${}^{c}$	5.412 ${}^{c}$	8.198 ${}^{c}$	2.96 ${}^{c}$	5.809 ${}^{c}$

The means in the columns followed by the same letters are not significantly different (*p* < 0.05) according to unprotected Fisher’s Least Significant Difference (LSD) test. In addition, values are displayed on the log scale.

## Data Availability

Not applicable.
